# The Value of Ursodeoxycholic Acid and Mesenchymal Stem Cells in the Treatment of Severe COVID-19

**DOI:** 10.3390/microorganisms12071269

**Published:** 2024-06-22

**Authors:** Qi Zheng, Yuetong Li, Guoping Sheng, Lanjuan Li

**Affiliations:** 1 State Key Laboratory for Diagnosis and Treatment of Infectious Diseases, National Clinical Research Center for Infectious Diseases, National Medical Center for Infectious Diseases, Collaborative Innovation Center for Diagnosis and Treatment of Infectious Diseases, The First Affiliated Hospital, Zhejiang University School of Medicine, 79 Qingchun Rd., Hangzhou 310003, China; 22218339@zju.edu.cn (Q.Z.); 22218871@zju.edu.cn (Y.L.); 2 Department of Infectious Disease, Shulan (Hangzhou) Hospital Affiliated to Zhejiang Shuren University, Shulan International Medical College, Hangzhou 310022, China

**Keywords:** UDCA, MSCs, COVID-19, prognosis

## Abstract

**Objective:** The objective of this study was to evaluate the therapeutic efficacy of ursodeoxycholic acid (UDCA) and mesenchymal stem cells (MSCs) in patients with severe COVID-19. **Methods:** We included severe COVID-19 patients hospitalized at Shulan (Hangzhou) Hospital between December 2022 and June 2023. We used a logistic regression model to compare the use of UDCA and MSCs in the two distinct groups of improved and poor outcomes. It is noteworthy that the deterioration group encompassed instances of both death and abandonment of treatment. The receiver operating characteristic (ROC) curve was plotted to assess the performance of the model. The aim was to assess the therapeutic effect of UDCA and MSCs on the outcome of severe COVID-19 patients. **Results:** A total of 167 patients with severe COVID-19 were included in this study. The analysis revealed that out of 42 patients (25.1%), 17 patients (10.2%) had taken UDCA, and 17 patients (10.2%) had used MSCs. Following a multivariable logistic regression, the results indicated a negative association between UDCA treatment (OR = 0.38 (0.16–0.91), *p* = 0.029), MSCs treatment (OR = 0.21 (0.07–0.65), *p* = 0.007), and the risk of severe COVID-19 mortality. Additionally, age showed a positive association with the risk of mortality (OR = 1.03 (1.01–1.07), *p* = 0.025). **Conclusions:** UDCA and MSCs have shown potential in improving the prognosis of severe COVID-19 patients and could be considered as additional treatments for COVID-19 in the future.

## 1. Introduction

Since the epidemic, SARS-CoV-2 has attracted significant attention due to its high infectivity and substantial mortality rate. According to the WHO, as of September 2023, global SARS-CoV-2 infections have surpassed 770 million, with a death toll of approximately 6.95 million, imposing an immense burden on the global healthcare system.

COVID-19 vaccines, novel antivirals, and plasma exchange have demonstrated efficacy in improving the prognosis of patients with COVID-19 to a certain extent [1,2,3]. Nevertheless, the droplet transmission of SARS-CoV-2, coupled with its rapid mutation rate, makes the treatment and control of novel coronavirus pneumonia more difficult [4].

Currently, although novel antivirals such as Paxlovid and Molnupiravir have been developed for COVID-19, some research suggests that these medications may not effectively reduce the mortality rate in severe cases of COVID-19. In addition, the timing of antiviral use remains controversial, as the benefits of using antivirals in the later stages of disease progression may not be significant [5,6,7]. Additionally, antivirals may have side effects such as liver damage and neurological reactions, and their use in combination with other medications may potentially increase treatment risks [8,9,10]. Therefore, there is an imperative need to discover novel and effective therapies for COVID-19.

Ursodeoxycholic acid (UDCA), a hydrophilic bile acid, has been primarily used for the treatment of cholestatic liver diseases, such as primary biliary cirrhosis [11]. As a farnesoid X receptor (FXR) inhibitor, UDCA has been shown to improve the prognosis of patients with COVID-19 by reducing the expression of angiotensin-converting enzyme 2 (ACE2), a viral host receptor, by inhibiting the FXR activity [12]. However, the role of UDCA has been controversial, with a retrospective study showing that UDCA can reduce the risk of SARS-CoV-2 infection and the risk of exacerbation [13], while Francesca et al. argued that there was a lack of effective evidence for UDCA improving COVID-19 prognosis [14].

MSCs, originating from the mesoderm and ectoderm of the early embryo, possess robust tissue repair and immunomodulatory abilities through the secretion of soluble paracrine protein factors and exosomes [15]. A clinical study suggested that SMCs were able to significantly reduce mortality in patients with acute respiratory distress symptoms induced by the H7N9 virus [16]. Furthermore, A retrospective study showed that MSCs also play a role in the amelioration of lung inflammation in COVID-19 patients [17]. In addition, a meta-analysis concluded that MSCs were effective in reducing all-cause mortality in patients with COVID-19 [18].

However, few investigations have been conducted regarding the role of UDCA and MSCs in patients with severe COVID-19, and the efficacy of UDCA remains controversial [14]. Therefore, to further evaluate the therapeutic effects of UDCA and MSCs, this paper retrospectively analyzed patients with severe COVID-19 who were hospitalized in the Shulan (Hangzhou) Hospital from December 2022 to June 2023.

## 2. Methods

### 2.1. Study Design and Population

**Figure 1:**
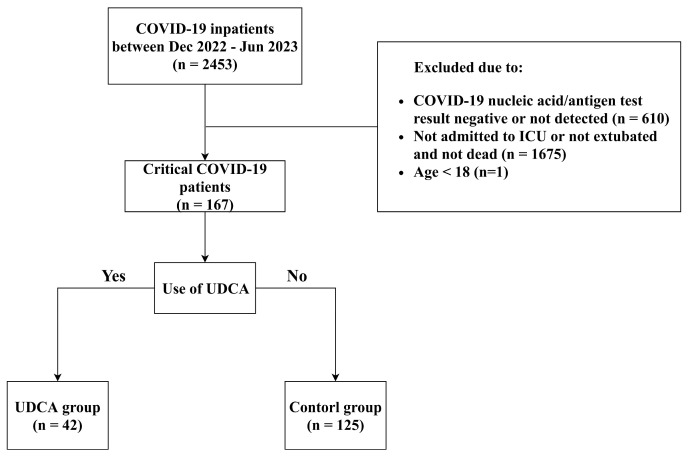
The flow chart shows the inclusion and exclusion criteria of severe COVID-19 inpatients in this study. ICU: intensive care unit; UDCA: ursodeoxycholic acid.

We conducted a single-center, retrospective, observational case-control study, which included 167 patients with severe COVID-19 admitted to Shulan (Hangzhou) Hospital (Zhejiang, China) from December 2022 to June 2023, where severe COVID-19 was defined as either admission to the Intensive Care Unit (ICU) or undergoing endotracheal intubation. The patients who received UDCA were categorized into the UDCA group (n = 42), while those who did not receive UDCA were categorized into the control group (n = 125), and the two groups will be compared (Figure 1).

### 2.2. Data Source

The data in this study were extracted from electronic medical records. Clinical information was collected for each patient including demographics (age, gender), clinical presentation on admission (fever, chill, cough, pharyngalgia, myalgia, unconsciousness, stomachache, nausea, vomiting, diarrhea, chest tightness), comorbidities (hypertension, diabetes, cancer, nervous system disease, chronic obstructive pulmonary disease), complete blood count, blood biochemistry, and treatment (glucocorticoid, antibiotics, antivirals, UDCA, MSCs, blood purification (including artificial liver treatment and continuous renal replacement therapy)), including antivirals such as Paxlovid, Molnupiravir, Azvudine, and VV116.

### 2.3. Statistical Analysis

We report variable values using percentages, medians, and interquartile ranges (IQR). Analyses were performed using SPSS27 and R (version 4.3.0). The Mann–Whitney test was used for variables not conforming to a normal distribution. The Chi-square test and chi-square correction test were used for dichotomous variables. Subsequently, we included variables with correlations with the prognosis of severe patients in the model and performed logistic regression, and the receiver operating characteristic (ROC) curves were meticulously constructed to evaluate the efficacy of the model. *p* < 0.05 was considered statistically significant.

## 3. Results

### 3.1. Patients Characteristics

This study included 167 patients with severe COVID-19 admitted to Shulan (Hangzhou) Hospital from December 2022 to June 2023 due to a SARS-CoV-2 infection. Among these patients, 131 patients (78.4%) died in the hospital or opted to discontinue the treatment, while 36 patients (21.6%) were discharged after hospitalization. Of all the patients included, a total of 42 patients (25.1%) received UDCA at a dose of approximately 10 mg/Kg per day in either tablet or capsule form. Seventeen patients (10.2%) received menstrual blood MSCs at an injectable dose of 3 × 10^7^ cells either through the transhepatic artery or peripheral vein. Table 1 delineates and compares the demographics, symptoms, comorbidities, inflammatory markers, blood biochemistry, and treatment of the UDCA group and control group.

The results show that there were no significant differences in age and gender between the UDCA group and the control group. The UDCA group reported fewer complaints of chest tightness upon admission compared to the control group. In terms of comorbidities, the proportion of cancer patients was lower and that of liver disease patients was higher in the UDCA group. In serologic testing, the levels of C-reactive protein (CRP) showed no significant statistical differences between the two groups, but the UDCA group had significantly lower serum creatinine levels upon admission compared to the control group. In terms of treatment, there were no significant statistical differences in the use of MSCs, glucocorticoids, antiviral drugs, antibiotics, antifungal drugs, and blood purification between the two groups.

At the same time, patients with severe COVID-19 were divided into an MSCs group and a control group according to whether MSCs were used to preliminarily analyze the correlation between MSCs treatment and patient outcomes (Table 2).

The results indicated no significant differences between the MSCs group and the control group in terms of age, gender, comorbidities, and symptoms. Notably, patients in the MSCs group had poorer liver function and a higher proportion of patients received blood purification therapy.

### 3.2. Results of Mortality Risk Analysis in Patients with Severe COVID-19

We then performed univariate logistic regression to further identify factors associated with the risk of death in patients with severe COVID-19 (Table 3).

Subsequently, we performed multivariate logistic regression analysis based on univariate logistic regression analysis (Table 4). The results indicated that the risk of death in patients with severe COVID-19 increased with age. Additionally, liver disease was a high-risk factor for mortality. The use of MSCs and UDCA was negatively correlated with the risk of death.

**Figure 2:**
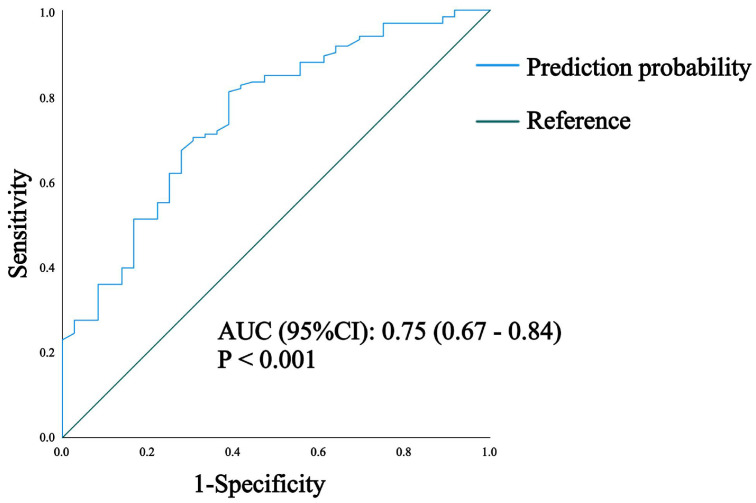
ROC curve of mortality risk prediction model. AUC: area under curve; CI: confidence interval.

We then plotted the ROC curve for predicting the risk of death. The area under curve (AUC) of ROC curve was 0.75 (0.67–0.84), *p* < 0.001, which indicated that the prediction model had a high test power; that is, the risk of death in patients with severe COVID-19 was statistically strongly correlated with age, liver disease, MSCs treatment, and UDCA treatment (Figure 2).

## 4. Discussion

COVID-19 is caused by SARS-CoV-2 through an interaction between the spiny protein (SP), the receptor-binding domain (RBD), and angiotensin-converting enzyme 2 (ACE2) [19]. The health problems it causes require continuous attention. Antivirals, such as Molnupiravir, a ribonucleoside analog, can target the RNA polymerase of SARS-CoV-2 and thus inhibit viral replication. However, the efficacy and safety of antivirals in the treatment of COVID-19 are still controversial, with some studies reporting that they can reduce the risk of hospitalization or death [20], while others have reported that they do not play a significant role in improving mortality in patients with severe disease [21]. On the other hand, the development of antiviral drug resistance will have a negative impact on the treatment of COVID-19, and it has been reported that Molnupiravir will promote a mutation in the SARS-CoV-2 genes and increase the risk of drug resistance [22]. The same situation exists among other antivirals; Nirmatrelvir and Pomotrelvir, as novel SARS-CoV-2 M^pro^ inhibitors, have been found to be more susceptible to the emergence of E166V mutant strains during prolonged use, leading to a high degree of drug resistance [23,24]. Therefore, novel, safe, and effective treatments are essential.

UDCA, as a hydrophilic amino acid, has been widely used in the clinical treatment of cholestatic liver disease with choleretic, litholytic, anti-inflammatory, and anti-apoptotic effects [25]. UDCA can down-regulate the expression level of the SARS-CoV-2 host receptor ACE2 in tissues by inhibiting the activity of FXR, which in turn reduces the susceptibility of the human body to SARS-CoV-2. Saleem Abdulrab suggested that UDCA could play a role in preventing or mitigating cytokine storms by inhibiting the inflammatory response [26]. Pham Xuan Thuy found that UDCA could inhibit the damage caused by the interaction between SP and ACE2 and promote the repair of the airway epithelium through basic research [19]. In a retrospective analysis of cirrhotic patients, Binu V John found that UDCA played a positive role in improving the severity of COVID-19 [13]. In contrast, Francesca Colapietro, in a single-center retrospective analysis, concluded that UDCA, while reducing the use of continuous positive pressure ventilation (CPAP), did not have a discernible impact on the amelioration of mortality [14].

MSCs are stromal cells with strong regenerative, tissue repair, and immunomodulatory abilities [27], and have been utilized in the treatment of liver diseases, vascular diseases, lung diseases, and various other diseases [28]. Giacomo Lanzoni conducted a clinical trial and found that MSCs had a positive significance in reducing cytokines and improving survival in patients with severe COVID-19, and demonstrated its safety in therapy [29]. Nevertheless, it is necessary to acknowledge that current research is still in its preliminary stage, and challenges exist in terms of quality management and the design of clinical trials pertaining to MSCs.

Our study analyzed the treatment of patients with severe COVID-19, who have a high mortality rate and require more attention. In our analysis, we included examinations such as blood biochemistry, coagulation function, and inflammatory markers, and co-analyzed the role of UDCA, MSCs, glucocorticoids, antibiotics, antivirals, ACEI and antifungal drugs in improving the outcomes of severe COVID-19 patients, and we found that UDCA and MSCs hold potential in ameliorating the outcomes of patients with severe COVID-19, reducing the mortality rate of patients. Notably, we purposely omitted the assessment of hospitalization duration, as well as the dose and start and stop times of antibiotics, antivirals, and antifungals in our analysis because these factors may be affected by clinical confounders, such as time of diagnosis, availability of ICU resources, and time to feedback of drug sensitivity results.

Our study has some limitations. Firstly, the number of cases of severe COVID-19 we included will be lower than the actual number of patients with severe COVID-19 due to the scarcity of ICU resources and the existence of situations such as patients refusing to be admitted to the ICU. In addition, this investigation is a retrospective study, which restricts the acquisition of extra information, such as the patients’ prior continuous medication usage and the duration of UDCA intake. Also, the timing of medication administration may be affected because a positive nucleic acid test for COVID-19 does not represent the precise time at which the disease initially developed. Nevertheless, our findings suggest a positive effect of UDCA and MSCs on the outcomes of patients with severe COVID-19. This suggests that UDCA and MSCs will help to ameliorate the prognosis and increase the survival rate in patients with severe COVID-19. In addition, the effect of the timing, optimal dose, and duration of the drugs on the prognosis of patients with severe COVID-19 may be worth further exploration.

## Figures and Tables

**Table 1:** Description of demographic and clinical features and treatment of UDCA group, control group, and total cohort.

Variable		Total (n = 167)	Control Group (n = 125)	UDCA Group (n = 42)	*p*-Value
Demographic Characteristics					
Age (years) (Median, IQR)		76.00 (65.00, 86.50)	77.00 (66.00, 87.00)	72.50 (63.00, 84.75)	0.420
Gender (n, %)					0.658
	Male	112 (67.07)	85 (68.00)	27 (64.29)	
	Female	55 (32.93)	40 (32.00)	15 (35.71)	
Symptoms (n, %)					
	Fever	86 (51.50)	65 (52.00)	21 (50.00)	0.822
	Chill	4 (2.40)	2 (1.60)	2 (4.76)	0.564
	Cough	79 (47.31)	62 (49.60)	17 (40.48)	0.306
	Pharyngalgia	8 (4.79)	7 (5.60)	1 (2.38)	0.669
	Myalgia	6 (3.59)	5 (4.00)	1 (2.38)	0.993
	Unconsciousness	26 (15.57)	21 (16.80)	5 (11.90)	0.499
	Stomachache	5 (2.99)	2 (1.60)	3 (7.14)	0.193
	Nausea	5 (2.99)	4 (3.20)	1 (2.38)	1.000
	Vomiting	6 (3.59)	5 (4.00)	1 (2.38)	0.993
	Diarrhea	2 (1.20)	2 (1.60)	0 (0.00)	1.000
	Chest Tightness	41 (24.55)	37 (29.60)	4 (9.52)	0.009
Comorbidities (n, %)					
	Hypertension	108 (64.67)	85 (68.00)	23 (54.76)	0.120
	Diabetes	68 (40.72)	54 (43.20)	14 (33.33)	0.260
	Cancer	48 (28.74)	30 (24.00)	18 (42.86)	0.019
	NSD	46 (27.54)	33 (26.40)	13 (30.95)	0.568
	Cardiovascular diease	33 (19.76)	26 (20.80)	7 (16.67)	0.561
	Nephrosis	39 (23.35)	31 (24.80)	8 (19.05)	0.446
	Hepatopathy	28 (16.77)	12 (9.60)	16 (38.10)	0.001
	COPD	16 (9.58)	13 (10.40)	3 (7.14)	0.751
CBC (Median, IQR)					
	Neutrophil Ratio (%)	86.90 (77.55, 91.40)	88.10 (79.00, 92.00)	83.80 (76.95, 90.38)	0.055
	Lymphocyte Ratio (%)	7.00 (4.05, 13.40)	7.00 (3.90, 13.10)	7.45 (4.28, 15.55)	0.319
	Monocyte Ratio (%)	5.20 (2.55, 8.40)	4.80 (2.40, 8.10)	5.85 (3.50, 9.88)	0.025
	Eosinophil Ratio (%)	0.00 (0.00, 0.20)	0.00 (0.00, 0.10)	0.20 (0.00, 0.95)	0.001
	Basophil Ratio (%)	0.20 (0.10, 0.30)	0.20 (0.10, 0.20)	0.20 (0.10, 0.40)	0.202
	Neutrophil (10E9/L)	6.20 (3.70, 9.05)	6.60 (3.80, 9.30)	5.25 (2.80, 8.05)	0.040
	Lymphocyte (10E9/L)	0.50 (0.30, 0.80)	0.50 (0.30, 0.80)	0.50 (0.30, 0.88)	0.931
	Monocyte (10E9/L)	0.40 (0.20, 0.60)	0.40 (0.20, 0.60)	0.40 (0.20, 0.67)	0.463
	Eosinophil (10E9/L)	0.00 (0.00, 0.01)	0.00 (0.00, 0.01)	0.01 (0.00, 0.05)	0.001
CRP (mg/L) (Median, IQR)		70.60 (21.55, 127.05)	77.40 (25.00, 141.60)	45.30 (18.62, 93.73)	0.070
Biochemistry (Median, IQR)					
	ALT (U/L)	26.00 (16.00, 41.50)	25.00 (15.00, 42.00)	27.00 (18.00, 41.00)	0.535
	AST (U/L)	35.00 (23.00, 60.50)	36.00 (23.00, 63.00)	34.00 (20.50, 55.75)	0.527
	AST/ALT ratio	1.50 (1.00, 2.30)	1.60 (1.00, 2.30)	1.50 (1.02, 2.18)	0.701
	Serum creatinine (μmol/L)	100.00 (63.00, 194.00)	119.00 (71.00, 212.00)	66.50 (55.25, 100.00)	0.001
Treatment (n, %)					
	MSCs	17 (10.18)	14 (11.20)	3 (7.14)	0.647
	Glucocorticoids	150 (89.82)	112 (89.60)	38 (90.48)	1.000
	Antibiotics	164 (98.20)	122 (97.60)	42 (100.00)	0.573
	Antivirals	65 (38.92)	48 (38.40)	17 (40.48)	0.811
	Antifungal Drugs	108 (64.67)	81 (64.80)	27 (64.29)	0.952
	Probiotics	68 (40.72)	50 (40.00)	18 (42.86)	0.744
	Blood purification	96 (57.49)	72 (57.60)	24 (57.14)	0.791

UDCA: ursodeoxycholic acid; IQR: inter quartile range; NSD: nervous system disease; COPD: chronic obstructive pulmonary disease; CRP: c reactive protein; AST: aspartate aminotransferase; ALT: alanine aminotransferase; MSCs: mesenchymal stem cell.

**Table 2:** Description of demographic and clinical features and treatment of MSCs group, control group, and total cohort.

Variable		Total (n = 167)	Control Group (n = 150)	MSCs Group (n = 17)	*p*-Value
Demographic Characteristics					
Age (years) (Median, IQR)		76.00 (65.00, 86.50)	76.00 (63.25, 87.00)	77.00 (71.00, 81.00)	0.695
Gender (n, %)					0.744
	Male	112 (67.07)	100 (66.67)	12 (70.59)	
	Female	55 (32.93)	50 (33.33)	5 (29.41)	
Symptoms (n, %)					
	Fever	86 (51.50)	77 (51.33)	9 (52.94)	0.900
	Chill	4 (2.40)	3 (2.00)	1 (5.88)	0.352
	Cough	79 (47.31)	68 (45.33)	11 (64.71)	0.129
	Pharyngalgia	8 (4.79)	8 (5.33)	0 (0.00)	1.000
	Myalgia	6 (3.59)	4 (2.67)	2 (11.76)	0.115
	Unconsciousness	26 (15.57)	26 (17.33)	0 (0.00)	0.130
	Stomachache	5 (2.99)	5 (3.33)	0 (0.00)	1.000
	Nausea	5 (2.99)	4 (2.67)	1 (5.88)	0.419
	Vomiting	6 (3.59)	6 (4.00)	0 (0.00)	1.000
	Diarrhea	2 (1.20)	2 (1.33)	0 (0.00)	1.000
	Chest Tightness	41 (24.55)	39 (26.00)	2 (11.76)	0.320
Comorbidities (n, %)					
	Hypertension	108 (64.67)	97 (64.67)	11 (64.71)	0.997
	Diabetes	68 (40.72)	59 (39.33)	9 (52.94)	0.279
	Cancer	48 (28.74)	43 (28.67)	5 (29.41)	1.000
	NSD	46 (27.54)	42 (28.00)	4 (23.53)	0.917
	Cardiovascular diease	33 (19.76)	31 (20.67)	2 (11.76)	0.581
	Nephrosis	39 (23.35)	36 (24.00)	3 (17.65)	0.776
	Hepatopathy	28 (16.77)	26 (17.33)	2 (11.76)	0.810
	COPD	16 (9.58)	14 (9.33)	2 (11.76)	1.000
CBC (Median, IQR)					
	Neutrophil Ratio (%)	86.90 (77.55, 91.40)	86.85 (77.12, 91.40)	90.00 (85.40, 92.30)	0.155
	Lymphocyte Ratio (%)	7.00 (4.05, 13.40)	7.10 (4.12, 14.30)	5.40 (3.40, 7.40)	0.170
	Monocyte Ratio (%)	5.20 (2.55, 8.40)	5.25 (2.52, 8.47)	5.00 (2.70, 7.80)	0.477
	Eosinophil Ratio (%)	0.00 (0.00, 0.20)	0.00 (0.00, 0.20)	0.00 (0.00, 0.30)	0.974
	Basophil Ratio (%)	0.20 (0.10, 0.30)	0.20 (0.10, 0.30)	0.20 (0.10, 0.20)	0.757
	Neutrophil (10E9/L)	6.20 (3.70, 9.05)	6.05 (3.52, 8.88)	6.60 (5.80, 10.80)	0.072
	Lymphocyte (10E9/L)	0.50 (0.30, 0.80)	0.50 (0.30, 0.88)	0.50 (0.40, 0.80)	0.934
	Monocyte (10E9/L)	0.40 (0.20, 0.60)	0.35 (0.20, 0.60)	0.50 (0.30, 0.60)	0.280
	Eosinophil (10E9/L)	0.00 (0.00, 0.01)	0.00 (0.00, 0.01)	0.00 (0.00, 0.03)	0.690
CRP (mg/L)(Median, IQR)		70.60 (21.55, 127.05)	68.50 (22.30, 130.47)	70.60 (19.70, 107.00)	0.564
Biochemistry (Median, IQR)					
	ALT (U/L)	26.00 (16.00, 41.50)	25.00 (15.25, 40.00)	47.00 (23.00, 57.00)	0.048
	AST (U/L)	35.00 (23.00, 60.50)	35.00 (23.00, 60.75)	37.00 (17.00, 54.00)	0.721
	AST/ALT ratio	1.50 (1.00, 2.30)	1.60 (1.10, 2.40)	1.00 (0.70, 1.30)	0.001
	Serum creatinine (μmol/L)	100.00 (63.00, 194.00)	100.50 (63.00, 199.50)	84.00 (65.00, 139.00)	0.529
Treatment (n, %)					
	UDCA	42 (25.15)	39 (26.00)	3 (17.65)	0.647
	Glucocorticoids	150 (89.82)	133 (88.67)	17 (100.00)	0.298
	Antibiotics	164 (98.20)	147 (98.00)	17 (100.00)	1.000
	Antivirals	65 (38.92)	55 (36.67)	10 (58.82)	0.076
	Antifungal Drugs	108 (64.67)	95 (63.33)	13 (76.47)	0.283
	Probiotics	68 (40.72)	58 (38.67)	10 (58.82)	0.109
	Blood purification	96 (57.49)	79 (52.67)	17 (100)	0.001

UDCA: ursodeoxycholic acid; IQR: inter quartile range; NSD: nervous system disease; COPD: chronic obstructive pulmonary disease; CRP: c reactive protein; AST: aspartate aminotransferase; ALT: alanine aminotransferase.

**Table 3:** Results of death risk analysis in univariate logistic regression.

Variable	Beta	SE	*p*-Value	OR (95%CI)	RR (95%CI)
Age	0.04	0.01	0.003	1.04 (1.01–1.07)	/
CRP	0.00	0.00	0.250	1.00 (1.00–1.01)	/
Serum creatinine	0.00	0.00	0.204	1.00 (1.00–1.00)	/
ALT	0.01	0.01	0.444	1.01 (0.99–1.02)	
Sex					1.151 (0.633–2.094)
Female				1.00 (Reference)	
Male	0.18	0.39	0.647	1.20 (0.55–2.59)	
Liver disease					1.612 (0.619–4.196)
No				1.00 (Reference)	
Yes	0.58	0.58	0.310	1.79 (0.58–5.55)	
Use of MSCs					0.397 (0.217–0.726)
No				1.00 (Reference)	
Yes	−1.35	0.53	0.011	0.26 (0.09–0.73)	
Use of UDCA					0.528 (0.298–0.935)
No				1.00 (Reference)	
Yes	−0.85	0.40	0.035	0.43 (0.19–0.94)	
Use of antibiotics					1.562 (0.307–7.948)
No				1.00 (Reference)	
Yes	0.61	1.24	0.622	1.84 (0.16–20.92)	
Use of antivirals					0.406 (0.224–0.734)
No				1.00 (Reference)	
Yes	−1.17	0.39	0.003	0.31 (0.15–0.67)	
Use of antifungal drugs					0.805 (0.427–1.519)
No				1.00 (Reference)	
Yes	−0.27	0.40	0.499	0.76 (0.34–1.68)	
Use of probiotics					0.962 (0.535–1.728)
No				1.00 (Reference)	
Yes	−0.05	0.38	0.896	0.95 (0.45–2.01)	
Use of blood purification					0.890 (0.474–1.561)
No				1.00 (Reference)	
Yes	−0.19	0.38	0.620	0.83 (0.39–1.76)	

UDCA: ursodeoxycholic acid; OR: odds ratio; CI: confidence interval; CRP: c reactive protein; MSCs: mesenchymal stem cells.

**Table 4:** Results of death risk analysis in multivariate logistic regression.

Variable	Beta	SE	*p*-Value	OR (95%CI)
Age	0.03	0.02	0.025	1.03 (1.01–1.07)
Use of MSCs				
No				1.00 (Reference)
Yes	−1.57	0.58	0.007	0.21 (0.07–0.65)
Use of UDCA				
No				1.00 (Reference)
Yes	−1.46	0.50	0.029	0.38 (0.16–0.91)
Use of antivirals				
No				1.00 (Reference)
Yes	−0.73	0.44	0.097	0.48 (0.20–1.14)

UDCA: ursodeoxycholic acid; OR: odds ratio; CI: confidence interval; MSCs: mesenchymal stem cells.

## Data Availability

The datasets generated or analyzed during this study are available from the corresponding author on reasonable request.
